# Changes in Uric Acid Levels following Bariatric Surgery Are Not Associated with *SLC2A9* Variants in the Swedish Obese Subjects Study

**DOI:** 10.1371/journal.pone.0051658

**Published:** 2012-12-14

**Authors:** Mark A. Sarzynski, Peter Jacobson, Tuomo Rankinen, Björn Carlsson, Lars Sjöström, Claude Bouchard, Lena M. S. Carlsson

**Affiliations:** 1 Human Genomics Laboratory, Pennington Biomedical Research Center, Baton Rouge, Louisiana, United States of America; 2 Institutes of Medicine, The Sahlgrenska Academy, University of Gothenburg, Gothenburg, Sweden; Innsbruck Medical University, Austria

## Abstract

**Context and Objective:**

Obesity and *SLC2A9* genotype are strong determinants of uric acid levels. However, data on *SLC2A9* variants and weight loss induced changes in uric acid levels are missing. We examined whether the changes in uric acid levels two- and ten-years after weight loss induced by bariatric surgery were associated with *SLC2A9* single nucleotide polymorphisms (SNPs) in the Swedish Obese Subjects study.

**Methods:**

SNPs (N = 14) identified by genome-wide association studies and exonic SNPs in the *SLC2A9* gene locus were genotyped. Cross-sectional associations were tested before (N = 1806), two (N = 1664) and ten years (N = 1201) after bariatric surgery. Changes in uric acid were compared between baseline and Year 2 (N = 1660) and years 2 and 10 (N = 1172). A multiple testing corrected threshold of P = 0.007 was used for statistical significance.

**Results:**

Overall, 11 of the 14 tested *SLC2A9* SNPs were significantly associated with cross-sectional uric acid levels at all three time points, with rs13113918 showing the strongest association at each time point (R^2^ = 3.7−5.2%, 3.9×10^−22^≤p≤7.7×10^−11^). One SNP (rs737267) showed a significant association (R^2^ = 0.60%, P = 0.002) with change in uric acid levels from baseline to Year 2, as common allele homozygotes (C/C, N = 957) showed a larger decrease in uric acid (−61.4 µmol/L) compared to minor allele carriers (A/X: −51.7 µmol/L, N = 702). No SNPs were associated with changes in uric acid from years 2 to 10.

**Conclusions:**

SNPs in the *SLC2A9* locus contribute significantly to uric acid levels in obese individuals, and the associations persist even after considerable weight loss due to bariatric surgery. However, we found little evidence for an interaction between genotype and weight change on the response of uric acid to bariatric surgery over ten years. Thus, the fluctuations in uric acid levels among the surgery group appear to be driven by the weight losses and gains, independent of *SLC2A9* genotypes.

## Introduction

Chronic hyperuricemia is the underlying cause of gout, the most prevalent inflammatory arthritis in developed countries [1]. Furthermore, elevated serum uric acid levels are associated with an increased risk of mortality and cardiovascular disease, as well as cardiovascular disease risk factors such as obesity, hypertension, dyslipidemia, diabetes, and the metabolic syndrome [2], [3]. Elevated serum uric acid levels are associated with obesity, particularly visceral adiposity, via both increased production and decreased renal excretion of urate. [4]–[6] In the Turkish Adult Risk Factor Study, abdominal obesity was the strongest determinant of uric acid concentration variability after adjustment for 13 variables including total cholesterol, triglycerides, C-reactive protein, and blood pressure [7]. Several intervention and prospective studies suggest that changes in weight and obesity measures may play a role in the regulation of serum uric acid levels [6], [8]–[12], although the reverse scenario may also be possible [13]. For example, in the Coronary Artery Risk Development in young Adults (CARDIA) Study, 10-year changes in body mass index (BMI) and waist circumference were positively and significantly associated with 10-year change in uric acid [12].

Bariatric surgery has been considered the most reliable method of achieving long term weight loss. However, few studies have reported on the longitudinal changes in uric acid levels after bariatric surgery [14]–[16]. In the Swedish Obese Subjects (SOS) study, serum uric acid levels decreased 15% and 6% two and ten years after bariatric surgery, respectively [16]. In SOS, the surgery group had lower incidence rates of hyperuricemia and higher recovery from hyperuricemia over two and ten years compared to their matched, conventionally treated obese controls [16]. However, there was large inter-individual variation in the changes of uric acid levels to weight loss achieved through bariatric surgery in SOS subjects.

It is well known that uric acid levels are influenced by a strong genetic component, with heritability estimates ranging from 0.25–0.73 [17]–[19]. In recent genome-wide association studies (GWASs) of serum uric acid concentrations [20]–[25], the strongest associations in subjects of both European and African American descent have been found for single nucleotide polymorphisms (SNPs) in the solute carrier family 2, member 9 (*SLC2A9*) gene on chromosome 4, encoding a putative glucose transporter. This protein functions as a high-capacity urate transporter in humans [24], [26]. A meta-analysis of 14 GWAS reports totaling 28,141 subjects of European descent found the strongest association with serum uric acid levels at the *SLC2A9* locus (rs734553, p = 5.2×10^−201^), with 788 SNPs in *SLC2A9* reaching the genome-wide significance threshold of 5×10^−8^ [27]. These associations have been found to be sex-specific, with genotype having a greater effect on lowering uric acid levels in women compared to men [22], [27]. Furthermore, the sex-specific associations of *SLC2A9* variants with uric acid levels have been found to be modified by BMI, with stronger effect sizes in subjects with high BMI [28].

Thus, several *SLC2A9* SNPs have been reported to be associated with uric acid levels and there are known increases in uric acid with weight gain and decreases with weight loss. However, no study has examined the association of *SLC2A9* variants with uric acid levels after sustained weight loss obtained through a lifestyle or surgical intervention. Thus, the purpose of this study is to determine whether the changes in uric acid observed with weight fluctuations are conditioned by DNA sequence differences at the *SLC2A9* locus. For this purpose, we tested the association of GWAS-derived and exonic SNPs in *SLC2A9* with serum uric acid levels up to 10 years after bariatric surgery in patients of the SOS Study.

## Methods

### Ethics Statement

Written Informed consent was obtained for all study participants. All clinical investigations have been conducted according to the principles expressed in the Declaration of Helsinki. The SOS study protocol was approved by the following Swedish ethics committees: Regional Institutional Review Board of Gothenburg University, Regional Institutional Review Board of Linköping University, Regional Institutional Review Board of Lund University, Regional Institutional Review Board of Karolinska Intitute, in Stockholm, Regional Institutional Review Board of Umeå University, Regional Institutional Review Board of Örebro University and Regional Institutional Review Board of Uppsala University. The SOS trial has been registered in the ClinicalTrials.gov registry (NCT01479452, http://clinicaltrials.gov/ct2/show/NCT01479452?term).

### Design of the SOS Study

The SOS study has previously been described in detail. [16] In brief, SOS is a prospective, non-randomized clinical trial of the health effects of intentional weight reduction in the severely obese. Inclusion criteria included age (37–60 years at accrual) and BMI (≥34 kg/m^2^ for males and ≥38 kg/m^2^ for females). Exclusion criteria, described elsewhere [29], were minimal and aimed at ensuring subjects in the surgery group could tolerate the operation. Between 1987 and 2001, a total of 4047 severely obese subjects were included from the registry study and from waiting lists at surgical departments. Among those, 2010 eligible subjects desiring surgery constituted the surgery group, whereas the matched control group of 2037 subjects was offered conventional treatment at their primary health-care center. Surgical treatment included vertical banded gastroplasty (n = 1368), banding (n = 377), or gastric bypass (n = 265). Baseline examinations took place 4 weeks before surgery and the intervention study began on the day of the surgically treated subject’s operation. Follow-up examinations (at 0.5, 1, 2, 3, 4, 6, 8, and 10 years) were calculated in relation to the date of surgery.

#### Study sample

For the present study, SOS version I DNA samples were used, which excludes data from 48 surgery patients affected by errors that occurred during plating of DNA samples. The present study sample was comprised of subjects from the surgical treatment group only with valid genotype and uric acid concentration data (N = 1806).

### Uric Acid Assessment

Serum uric acid measurements were performed at the baseline and years 2 and 10 follow-up examinations. Blood samples were collected after an overnight (10–12 hour) fast. Uric acid concentration was measured using a calorimetric uricase method on a Clinicon Prisma instrument (from 1987), a Technicon DAX 72 autoanalyzer (from 1991), a Beohringer Mannheim Hitachi 917 analyzer (from 1995), a Roche Diagnostics Modular system (from 2002) or a Roche Diagnostics Cobas 8000 system (from 2010).

### SNP Selection

SNPs from the *SLC2A9* gene were selected for the present study because they were identified in previous uric acid GWAS reports or located in exons. The following eight SNPs were derived from the first two GWAS reports [24], [25]: rs13129697, rs737267, rs4447863, rs7442295, rs13131257, rs6449213, rs1014290, and rs9291642 (the latter tags rs733175 for which the assay failed). Six *SLC2A9* SNPs were selected because they were exonic: rs2280205, rs734553 (tags rs16890979 [Val282Ile]), rs3733591, rs13125646, rs13113918, and rs6820230. Eight additional SNPs were genotyped as tagSNPs in case of assay failure: rs734553, rs6855911, rs4697701, rs16868246, rs13125209, rs13115193, rs7680126, and rs3796834.

### Genotyping

The SNPs were genotyped using Illumina (San Diego, CA) GoldenGate chemistry and Sentrix Array Matrix technology on the BeadStation 500GX. Genotype calling was done with the Illumina BeadStudio software and each call was confirmed manually. For quality control purposes, five CEPH control DNA samples (NA10851, NA10854, NA10857, NA10860, NA10861; all samples included in the HapMap Caucasian panel) were genotyped in triplicate. Concordance between the replicates as well as with genotypes from the HapMap database was 100%.

### Statistical Analysis

Hardy-Weinberg equilibrium was tested by comparing observed genotype frequencies to those expected based on the allele frequencies of each marker using the ALLELE procedure in SAS version 9.1 (SAS Institute Inc, Cary, NC). The pair-wise linkage disequilibrium (LD) among the SNPs was assessed using the ldmax program available in the GOLD software package. [30] Means and standard deviation (SD) were computed for all continuous variables at baseline, Year 2, and Year 10 in the total sample and by surgical technique. Differences in continuous and categorical variables between surgery groups were assessed using t-tests and chi-square tests, respectively.

Association models were performed using the total association model implemented in MERLIN version 1.1.2 [31]. This option uses a variance component model to estimate an additive effect for each SNP and carry out an association test. The evidence of association is evaluated by maximizing the likelihoods under two conditions: the null hypothesis (L0) restricts the additive genetic effect of the marker locus to zero (*b*a  = 0), whereas the alternative hypothesis does not impose any restrictions on *b*a. The quantity of twice the difference of the log likelihoods between the alternative and the null hypotheses (2[ln (L1) − ln (L0)]) is distributed as χ2 with 1 df (difference in number of parameters estimated).

Cross-sectional association models were performed at baseline, Year 2, and Year 10. The cross-sectional association models included age, sex, and body weight as covariates. Changes in uric acid levels over time were calculated from baseline to Year 2 (N = 1660) and years 2 and 10 (N = 1172). The uric acid change over time models included baseline age, sex, and percent change in weight between selected years (Δweight/initial weight) as covariates. Genotype effect size (R^2^) was defined as the proportion of total phenotypic variance explained by the genotype. Inclusion of surgery technique, lipid modifying medications, smoking, and hypertension and diabetes status as covariates in the association models did not change our overall results or interpretation. Concomitant anti-gout medication was not used as a covariate, as it was reported by less than 2 percent of the surgery cases. Thus, results are presented without these variables as covariates.

Multivariable regression models with forward selection were used to evaluate the contribution of nine predictor variables on the two changes in uric acid level after bariatric surgery phenotypes (Δuric acid from baseline to Year 2 and Δuric acid from years 2 and 10). Age, sex, smoking status, lipid medication status, diabetes status, surgical procedure, and change in HDL-C, triglycerides, and weight between the two time points were included in the models. To evaluate the ability of *SLC2A9* genotype to classify risk of hyperuricemia, we plotted receiver-operating-characteristic (ROC) curves for logistic regression models with and without *SLC2A9* rs13113918 genotype. The C statistic, a measure of the area under the ROC curve, was calculated with and without genotype. We used the prevalence of hyperuricemia at baseline, Year 2, and Year 10 as the outcome and included age, sex, weight, and hypertension status as covariates. Hyperuricemia was defined in SOS as uric acid levels ≥7.6 mg/dL or 450 µmol/L in men and ≥5.7 mg/dL or 340 µmol/L in women [16].

Since associations were tested with 14 SNPs, we applied a multiple testing correction as proposed by Nyholt [32]. Briefly, the method uses spectral decomposition of matrices of pairwise LDs (r) to estimate variance of eigenvalues. The effective number of independent SNPs for each candidate gene can be calculated based on the ratio of observed eigenvalue variance and its maximum. The effective number of SNPs can then be used to adjust the standard α level (e.g., 5%). Thus, in our study the corrected threshold for statistical significance was set to P = 0.007 (0.05/7) as the total effective number of SNPs was 7.

## Results

### Subject Characteristics

Table 1 shows the basic characteristics at baseline, Year 2, and Year 10 in all subjects and by surgical technique. Overall, mean weight loss was about 29 kg from baseline to Year 2 after surgery (weight loss period), whereas subjects regained an average of 7.3 kg of weight from Year 2 to Year 10 after surgery (weight regain period).

**Table 1:** Basic characteristics of SOS subjects with DNA and data for uric acid levels in the total sample and by surgery group.

Time		All subjects	Vertical banded gastroplasty	Banding	Gastric bypass	Main Effect	Post-hoc
Point	Variable	Mean (SD)	Mean (SD)	Mean (SD)	Mean (SD)	P value	P value
**Baseline**	**N**	1806	1213	345	248		
	**Age,** yrs	47.1 (5.9)	47.1 (5.9)	47.5 (6.0)	47.0 (6.0)	0.50	–
	**Sex,** % female	70.3	70.7	68.1	71.8	0.57	–
	**BMI,** kg/m^2^	42.3 (4.5)	42.3 (4.4)	41.6 (4.2)	43.8 (5.1)	<0.0001	<0.0001
	**Weight,** kg	120.9 (16.7)	120.2 (16.1)	119.9 (16.1)	125.3 (19.3)	<0.0001	<0.0001
	**Serum uric acid,** µmol/L	358.3 (78.6)	357.8 (76.6)	361.9 (80.9)	355.9 (84.5)	0.61	–
**Year 2**	**N**	1664	1105	328	231		
	**BMI,** kg/m^2^	32.3 (4.8)	32.7 (4.7)	32.5 (5.1)	29.9 (4.3)	<0.0001	<0.0001
	**ΔWeight from baseline,** kg	−28.6 (14.4)	−27.2 (12.8)	−25.4 (15.6)	−39.7 (14.6)	<0.0001	<0.0001
	**Serum uric acid,** µmol/L	300.1 (73.8)	303.3 (73.4)	302.4 (75.3)	281.5 (71.3)	0.0002	<0.0001
	**Δuric acid from baseline,** µmol/L	−58.2 (67.2)[N = 1660]	−55.0 (64.3)[N = 1101]	−58.1 (70.6)	−73.6 (74.0)	0.0006	0.0002
**Year 10**	**N**	1201	856	248	97		
	**BMI,** kg/m^2^	35.1 (5.7)	35.3 (5.6)	35.5 (6.1)	33.1 (4.9)	0.0007	0.0001
	**ΔWeight from Year 2,** kg	7.3 (13.4)	6.8 (13.1)	8.2 (15.4)	9.7 (9.9)	0.07	–
	**Serum uric acid,** µmol/L	329.8 (83.5)	330.8 (83.0)	331.4 (80.8)	317.7 (70.6)	0.33	–
	**Δuric acid from Year 2,** µmol/L	+28.7 (75.1)[N = 1172]	+27.7 (77.8)[N = 833]	+30.9 (67.1)[N = 244]	+32.2 (70.6)[N = 95]	0.76	

Main effect P value is for the main effect of surgery technique on variable of interest. For variables showing a significant main effect of surgery technique, post-hoc pair-wise comparisons were run to test the mean difference between the combined banding group (vertical banded gastroplasty and banding) and gastric bypass group. N represents the number of subjects with DNA and data for uric acid level at each time point. To convert µmol/L to mg/dL divide values by 59.48.

### Uric Acid Changes After Bariatric Surgery

**Figure 1:**
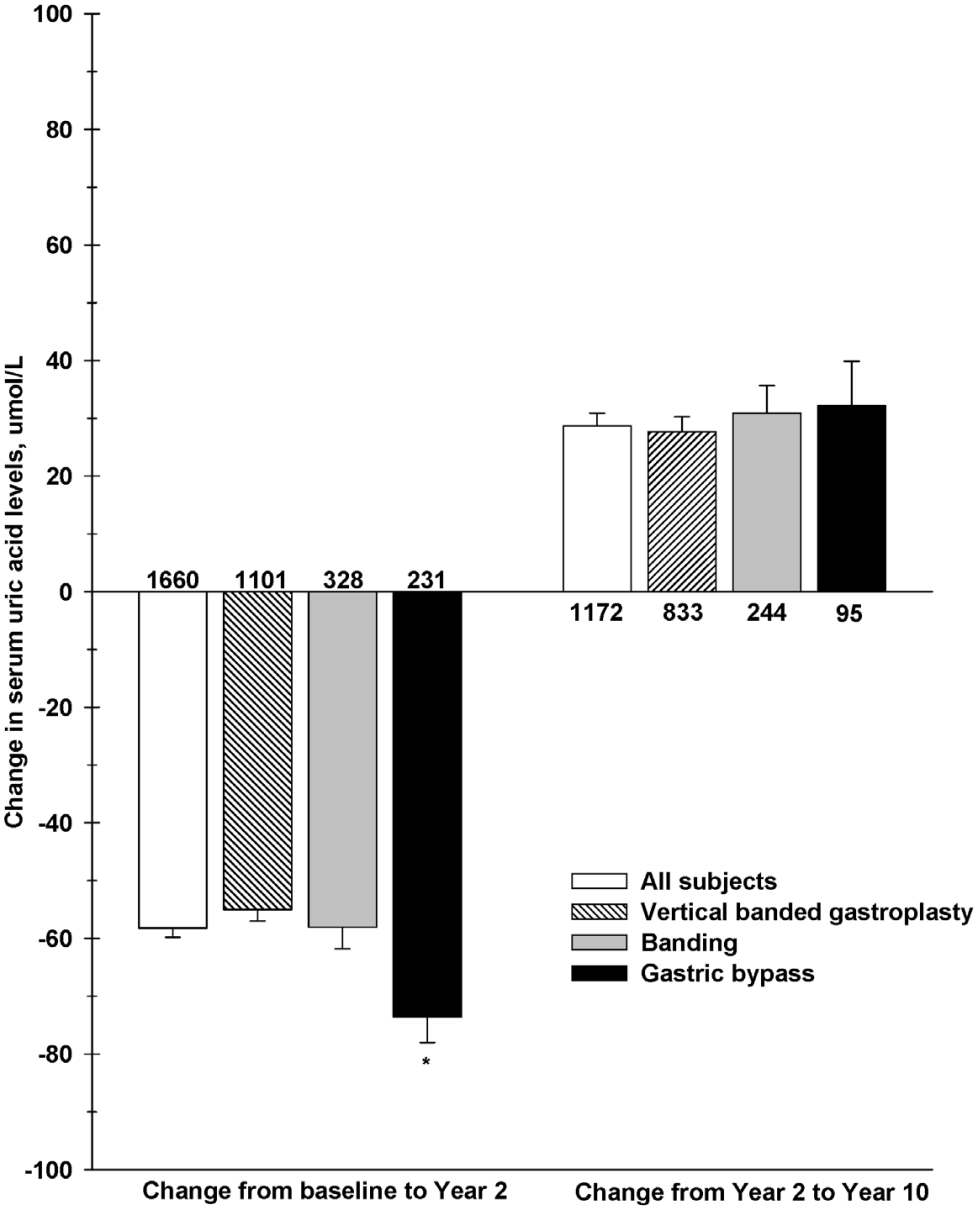
Mean changes in uric acid levels up to 10 years after bariatric surgery in SOS. The number of subjects in each group is indicated above or below each histogram bar. *P = 0.0002 for mean difference between gastric bypass and banding procedures groups.

Individual changes of uric acid during the weight loss period ranged from a decrease of 443.2 µmol/L to an increase of 220.5 µmol/L, and during the weight regain period ranged from a decrease of 484.1 µmol/L to an increase of 359.1 µmol/L. As shown in Figure 1 and Table 1, uric acid levels significantly decreased from baseline to Year 2 in all subjects, with the gastric bypass group showing a significantly larger mean decrease than the banding procedures groups, which was explained by the greater weight loss in this group (P = 0.12 when adjusting for change in weight). Conversely, uric acid levels increased by 28.7±75.1 µmol/L from Year 2 to Year 10 after surgery in all subjects, with no differences between surgical techniques (Table 1). There were no sex differences in the changes in uric acid levels from baseline to year 2. However, females had a significantly (P = 0.0005) larger mean increase (33.9±73.7 µmol/L) in uric acid levels from Year 2 to Year 10 compared to males (16.0±90.5 µmol/L).

### Factors Associated with Changes in Uric Acid

Change in weight was significantly correlated with change in uric acid levels from baseline to Year 2 (r  = 0.39, p<0.0001) and from Year 2 to Year 10 (r  = 0.34, p<0.0001)., Of the nine variables entered in the multivariable regression models, change in weight was the strongest predictor of change in uric acid levels after bariatric surgery (Supplementary Tables S1, S2, S3). Weight change from baseline to Year 2 explained 14.6% of the variance (β_coeff_  = 1.72, p<0.0001) in change in uric acid level during the same time period, while weight change from Year 2 to Year 10 explained 11.9% of the variance (β_coeff_  = 1.84, p<0.0001) in change in uric acid level during the same time period.

### Association of *SLC2A9* SNPs with Changes in Uric Acid

The minor allele frequencies, Hardy-Weinberg equilibrium, and pairwise LD among all included SNPs can be found in Supplementary Table S1, S3. All SNPs were in Hardy-Weinberg equilibrium, while several SNPs were in strong LD (r^2^>0.80). The associations of *SLC2A9* SNPs with changes in uric acid levels up to 10 years after bariatric surgery are shown in Table 2. Several SNPs showed nominal (p<0.05) associations with changes in uric acid levels from baseline to Year 2 after bariatric surgery, with only one remaining statistically significant after correcting for multiple testing. The *SLC2A9* rs737267 SNP showed a statistically significant association (R^2^ = 0.60%, P = 0.002) with change in uric acid levels from baseline to Year 2, as common allele homozygotes (C/C: −61.4 µmol/L, N = 957) showed a larger decrease in uric acid compared to minor allele carriers (A/C: −52.3 µmol/L, N = 603 and A/A: −48.2 µmol/L, N = 99). None of the SNPs were significantly associated with changes in uric acid from Year 2 to Year 10 after bariatric surgery (Table 2). The associations of *SLC2A9* SNPs with changes in uric acid levels for each surgical procedure group can be found in Supplementary Table S4.

**Table 2:** Associations between changes in serum uric acid levels and *SLC2A9* SNPs in SOS bariatric surgery patients when number of subjects has been maximized locally.

	Changes in uric acid					
*SLC2A9*	Baseline to Year 2			Year 2 to Year 10		
	N = 1660			N = 1172		
SNP	β	R^2^	p-value	β	R^2^	p-value
rs2280205	0.34	0.00%	0.88	4.83	0.24%	0.10
rs3733591	2.40	0.05%	0.37	−5.53	0.20%	0.13
rs734553	6.81	0.44%	0.008	−2.89	0.06%	0.41
rs13129697	4.57	0.21%	0.06	−2.55	0.05%	0.45
rs737267	7.78	0.59%	0.002	−4.56	0.16%	0.18
rs4447863	2.87	0.11%	0.18	−1.52	0.02%	0.60
rs7442295	6.98	0.42%	0.009	−0.06	0.00%	0.99
rs13131257	6.32	0.33%	0.02	−1.68	0.02%	0.65
rs13125646	6.26	0.33%	0.02	−1.74	0.02%	0.64
rs6449213	5.40	0.23%	0.05	−0.29	0.00%	0.94
rs13113918	6.44	0.36%	0.02	−3.66	0.09%	0.32
rs1014290	4.55	0.20%	0.08	−2.55	0.05%	0.47
rs9291642	4.35	0.11%	0.18	−1.86	0.02%	0.67
rs6820230	0.21	0.00%	0.93	−0.14	0.00%	0.96

All models are adjusted for age, sex, and percent change in body weight. β values represent change in changes in uric acid level (µmol/L) per copy of minor allele carried. To convert µmol/L to mg/dL divide values by 59.48.

### Cross-sectional *SLC2A9* SNP Associations

**Figure 2:**
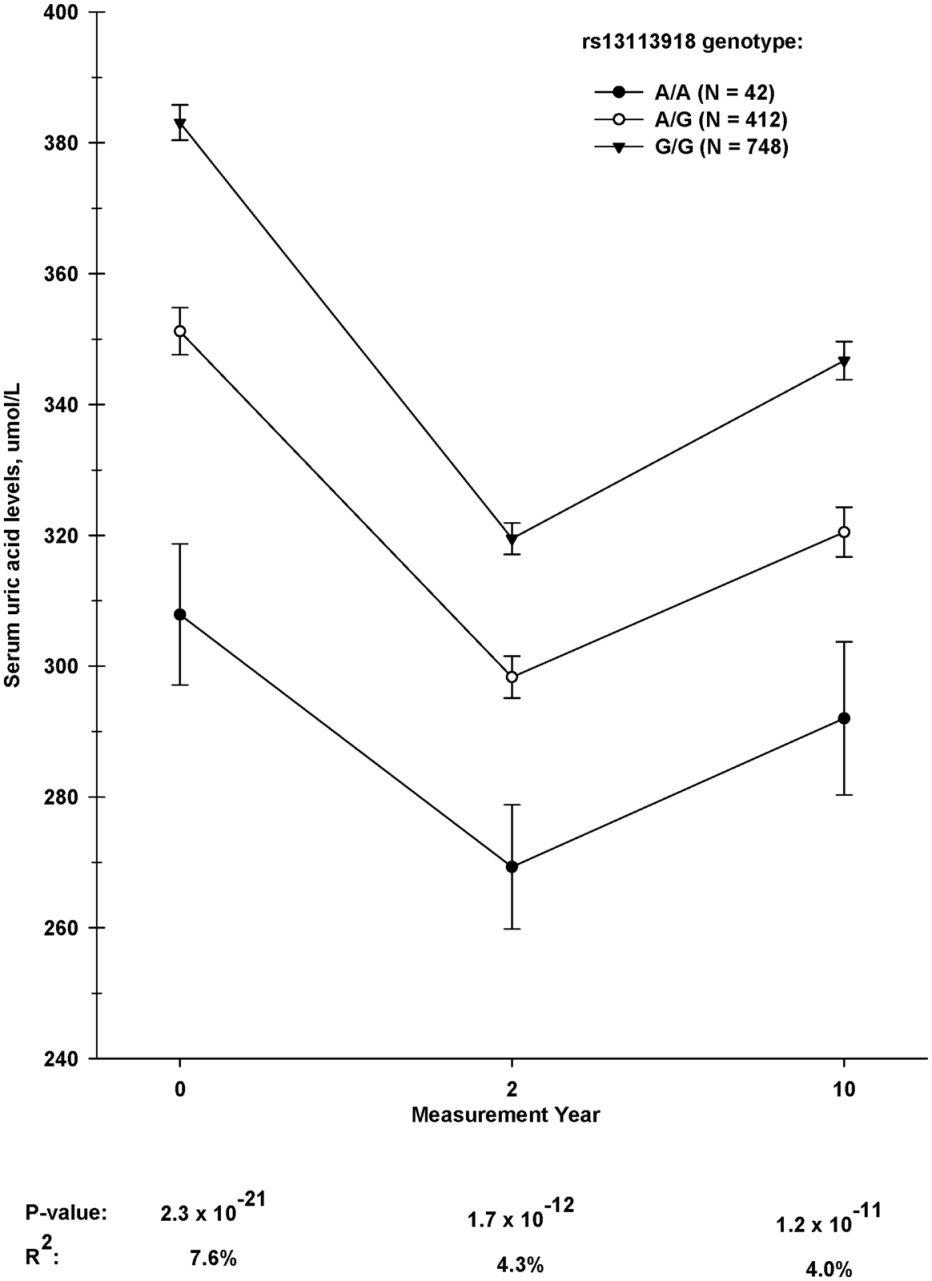
Uric acid levels at baseline and years 2 and 10 stratified by *SLC2A9* rs13113918 genotype. The number of subjects has been maximized globally (N = 1175 for all time points). Mean values adjusted for age, sex, and BMI. R^2^ is the proportion of total uric acid variance (%) explained by the SNP in the model.

The associations of *SLC2A9* SNPs with cross-sectional uric acid levels at baseline, Year 2, and Year 10 are shown in Table 3. After adjusting for covariates, 11 of the 14 tested SNPs were significantly associated with uric acid levels at all three time points, with *SLC2A9* SNP rs13113918 showing the strongest association at each time point (R^2^ = 3.7−5.2%, 3.9×10^−22^≤p≤7.7×10^−11^). As shown in Figure 2, the rank order of mean uric acid levels across rs13113918 genotype did not shift over ten years after bariatric surgery, as common allele homozygotes (G/G) had the highest mean uric acid levels followed by heterozygotes (A/G) and minor allele homozygotes (A/A) at all three time points. The cross-sectional association results for each surgical procedure group are presented in Supplementary Tables S5, S6, S7.

**Table 3:** Cross-sectional associations between serum uric acid levels and *SLC2A9* SNPs in SOS bariatric surgery patients when number of subjects has been maximized locally.

		Baseline			Year 2			Year 10	
*SLC2A9*		N = 1806			N = 1664			N = 1201	
SNP	β	R^2^	p-value	β	R^2^	p-value	β	R^2^	p-value
rs2280205	−6.09	0.36%	0.01	−5.78	0.43%	0.009	−2.72	0.07%	0.38
rs3733591	−0.17	0.00%	0.95	2.48	0.05%	0.36	−3.50	0.07%	0.37
rs734553	−26.65	4.98%	2.8×10−^21^	−21.31	4.15%	1.1×10^−16^	−20.88	2.76%	1.7×10^−8^
rs13129697	−23.91	4.34%	9.1×10^−19^	−20.60	3.98%	4.3×10^−16^	−21.12	3.06%	3.9×10^−9^
rs737267	−25.99	4.88%	2.0×10^−21^	−19.60	3.62%	5.2×10^−15^	−21.31	2.97%	2.7×10^−9^
rs4447863	−13.56	1.80%	1.3×10^−8^	−10.95	1.53%	5.0×10^−7^	−13.10	1.52%	1.9×10^−5^
rs7442295	−28.22	5.12%	2.8×10^−22^	−22.48	4.24%	3.0×10^−17^	−19.16	2.14%	5.7×10^−7^
rs13131257	−27.37	4.65%	2.5×10^−20^	−22.10	3.95%	4.0×10^−16^	−20.64	2.39%	1.2×10^−7^
rs13125646	−27.48	4.62%	3.3×10^−20^	−22.06	3.94%	4.3×10^−16^	−20.65	2.39%	1.2×10^−7^
rs6449213	−28.31	4.74%	3.0×10^−20^	−23.77	4.35%	2.6×10^−17^	−21.62	2.50%	7.8×10^−8^
rs13113918	−28.54	5.23%	3.9×10^−22^	−23.18	4.50%	9.6×10^−18^	−25.21	3.69%	7.7×10^−11^
rs1014290	−25.85	4.74%	3.7×10^−20^	−21.65	4.34%	3.6×10^−17^	−23.79	3.63%	1.7×10^−10^
rs9291642	−22.67	2.25%	1.9×10^−10^	−19.83	2.24%	1.2×10^−9^	−18.84	1.40%	5.1×10^−5^
rs6820230	−2.49	0.05%	0.34	−1.58	0.03%	0.51	−4.24	0.13%	0.20

All models are adjusted for age, sex, and body weight. β values represent change in cross-sectional uric acid level (µmol/L) per copy of minor allele carried. To convert µmol/L to mg/dL divide values by 59.48.

#### Prediction of prevalent hyperuricemia with and without *SLC2A9* rs13113918 genotype

The C statistic (area under the curve) for prevalence of hyperuricemia was 0.69 with and 0.66 without inclusion of rs13113918 genotype at baseline, 0.75 with and 0.73 without genotype at Year 2, and 0.76 with and 0.75 without genotype at Year 10 (Supplementary Figures S1, S2, S3).

### *SLC2A9* Genotype by Sex Interactions

We found evidence of genotype by sex interactions (tested using SNP rs13113918) on uric acid levels at baseline (P = 0.03), Year 2 (P = 0.03), and Year 10 (P = 0.001), as the genotype effect sizes were larger in females at all three time points. For example, in stratified analyses, the association of rs13113918 genotype with baseline uric acid levels was significant in both men (P = 0.0006) and women (P = 3.3×10^−21^), but the genotype effect size was three times larger in women (R^2^ = 7.0%) compared to men (R^2^ = 2.3%). No genotype by sex interactions (tested using SNP rs737267) were observed in either of the longitudinal change in uric acid models.

## Discussion

We examined the association of GWAS-derived and exonic *SLC2A9* variants with serum uric acid levels in severely obese subjects, who lost weight, up to ten years after bariatric surgery. Although one SNP showed an association with change in uric acid from baseline to Year 2 after bariatric surgery, no SNPs were associated with change in uric acid from years 2 to 10 after surgery. We found that the increases and decreases in uric acid levels were driven by the weight losses and gains; independent of *SLC2A9* genotypes, i.e. there was little evidence for an interaction between *SLC2A9* variants and weight change on changes in uric acid up to ten years after surgery. For example, the interaction between *SLC2A9* SNP rs13113918 genotype and Δweight on Δuric acid was not statistically significant from baseline to Year 2 (P = 0.04) or from Year 2 to Year 10 (P = 0.79). Thus, based on the top *SLC2A9* SNP, rs13113918, we can predict cross-sectional uric acid levels in obese individuals, as the rank order of mean uric acid levels across rs13113918 genotype did not shift following bariatric surgery. Furthermore, including *SLC2A9* rs13113918 genotype in logistic regression models improved the prediction of prevalent hyperuricemia at all three time points compared to models not including genotype. Therefore, our findings have potential clinical significance, as we can predict which individuals are predisposed to elevated uric acid levels using variation in the *SLC2A9* gene locus, even after lifestyle changes resulting in weight loss or weight gain.

*SLC2A9* encodes a transporter protein that belongs to class II of the facilitative glucose transporter family [33] and is thought to be both a urate and fructose transporter [34]. The role of *SLC2A9* variants on *SLC2A9* function is unknown. Thus, it is unknown how polymorphisms in *SLC2A9* may alter serum uric acid levels. A possible mechanism may be through modulation of renal excretion of uric acid, as polymorphisms of *SLC2A9* have also been shown to be associated with a low fractional excretion of uric acid [24]. In previous GWAS reports, the strongest associations were mapped to non-coding SNPs located near the 5′ end of the gene and within introns 3–7. The strongest cross-sectional associations in the present study were found with rs13113918, a coding SNP that gives rise to a synonymous substitution (Leu79Leu). Although ten coding SNPs have been reported, five of which give rise to nonsynonymous amino acid subsitutions, these SNPs showed less significant associations with serum uric acid than intronic SNPs in previous GWAS reports [24], [27]. This was also the case in the present study, as the three included nonsynonymous SNPs (rs2280205, rs3733591, rs6820230) showed no associations with cross-sectional uric acid levels or changes in uric acid over ten years.

Our results confirmed that uric acid levels varied closely with body weight and that SNPs in *SLC2A9* were associated with cross-sectional measures of uric acid, with sex-specific effects, as expected from the literature [20]–[25], [27]. The association of increased uric acid levels and obesity is well-established. We along with other studies [14], [15] have shown that sustained weight reduction, as a result of bariatric surgery, resulted in decreased serum uric acid levels in obese adults. The significant reduction in uric acid levels after bariatric surgery may be explained by a correction of uric acid renal clearance resulting from the near-normalization of hyperinsulinemia and insulin resistance in these subjects [6], [15], [35].

There is large inter-individual variation in uric acid levels regardless of weight status, which may be due to environmental and/or genetic differences. The present study found negligible evidence of interactions between *SLC2A9* variants and weight change on changes in uric acid up to ten years after bariatric surgery. There is a large body of evidence that shows when uric acid levels and body weight are assumed to be stable, *SLC2A9* genotype(s) can predict uric acid levels [20]–[25], [27]. However, when weight changes, uric acid levels also change and reach new steady state levels, independent of *SLC2A9* genotype. Thus, *SLC2A9* predicts the new cross-sectional uric acid level steady state, but does not appear to be involved in changes in uric acid levels due to weight loss, and presumably other metabolic changes induced by bariatric surgery. In other words, *SLC2A9* genotype does not appear to drive changes in uric acid levels when weight decreases or increases.

In conclusion, we found that weight changes were the driver of changes in uric acid levels, explaining less than 15% of the variance in uric acid level changes following bariatric surgery, with *SLC2A9* genotype accounting for a negligible proportion (<1%). Thus, a large portion of the variance is still unaccounted for. Our results indicate that *SLC2A9* variants had little effect on the inter-individual variation in the changes of uric acid in response to weight fluctuations induced by bariatric surgery. Although it appears that common *SLC2A9* variants do not contribute to uric acid changes in response to weight fluctuation, we cannot exclude other types of gene by weight interaction effects, as only 6% of the variation in serum uric acid can be accounted for by *SLC2A9* polymorphisms [22]. Therefore, further studies are needed that employ a genome-wide approach to identify the variants, outside of *SLC2A9*, associated with uric acid level changes in response to weight loss induced by bariatric surgery and/or lifestyle modifications.

## Supporting Information

Figure S1 **ROC curves for the prediction of prevalent hyperuricemia at baseline in SOS subjects.** The blue line represents the results when SLC2A9 rs13113918 genotype is included in the model, while the green line represents the results without genotype in the model.
(PDF)

Figure S2 **ROC curves for the prediction of prevalent hyperuricemia at Year 2 in SOS subjects.** The blue line represents the results when SLC2A9 rs13113918 genotype is included in the model, while the green line represents the results without genotype in the model.
(PDF)

Figure S3 **ROC curves for the prediction of prevalent hyperuricemia at Year 10 in SOS subjects.** The blue line represents the results when SLC2A9 rs13113918 genotype is included in the model, while the green line represents the results without genotype in the model.
(PDF)

Table S1 **Results of multivariate regression model with forward selection for predictors of change in uric acid from baseline to Year 2.**
(DOC)

Table S2 **Results of multivariate regression model with forward selection for predictors of change in uric acid from Year 2 to Year 10.**
(DOC)

Table S3 **SNP physical map locations, minor allele frequencies (MAF), Hardy-Weinberg equilibrium (HWE) test statistics, and pairwise linkage disequilibrium estimates (r2 below median, D’ above median) for SLC2A9 SNPs in SOS bariatric surgery patients.**
(DOC)

Table S4 **Associations between changes in serum uric acid levels and** ***SLC2A9*** **SNPs in SOS patients by surgical procedure.**
(DOC)

Table S5 **Cross-sectional associations between serum uric acid levels and SLC2A9 SNPs in SOS vertical banded gastroplasty patients when number of subjects has been maximized locally.**
(DOC)

Table S6 **Cross-sectional associations between serum uric acid levels and SLC2A9 SNPs in SOS banding patients when number of subjects has been maximized locally.**
(DOC)

Table S7 **Cross-sectional associations between serum uric acid levels and SLC2A9 SNPs in SOS gastric bypass patients when number of subjects has been maximized locally.**
(DOC)
