# Supplementary material for: Changes in Uric Acid Levels following Bariatric Surgery Are Not Associated with SLC2A9 Variants in the Swedish Obese Subjects Study
Source: PLoS One. 2012 Dec 14;7(12):e51658. doi: 10.1371/journal.pone.0051658 (PMC3522707; doi:10.1371/journal.pone.0051658)
Supplement: Figure S2 — ROC curves for the prediction of prevalent hyperuricemia at Year 2 in SOS subjects. The blue line represents the results when SLC2A9 rs13113918 genotype is included in the model, while the green line represents the results without genotype in the model. (PDF) [file pone.0051658.s002.pdf]

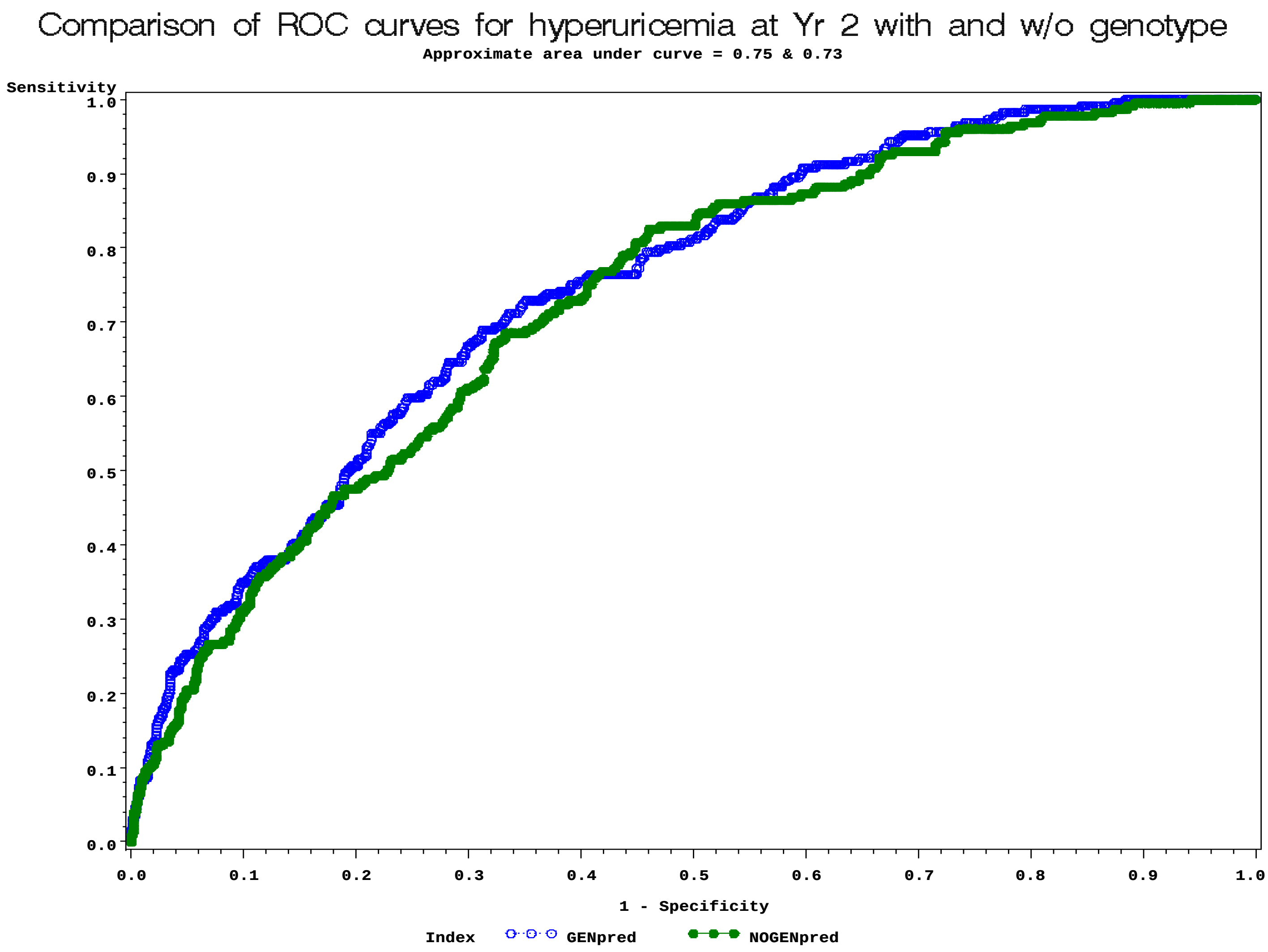

Figure S2. ROC curves for the prediction of prevalent hyperuricemia at Year 2 in SOS subjects with (blue line) and without (green line) the inclusion of SLC2A9 rs13113918 genotype in the model.
