# Supplementary material for: Changes in Uric Acid Levels following Bariatric Surgery Are Not Associated with SLC2A9 Variants in the Swedish Obese Subjects Study
Source: PLoS One. 2012 Dec 14;7(12):e51658. doi: 10.1371/journal.pone.0051658 (PMC3522707; doi:10.1371/journal.pone.0051658)
Supplement: Figure S3 — ROC curves for the prediction of prevalent hyperuricemia at Year 10 in SOS subjects. The blue line represents the results when SLC2A9 rs13113918 genotype is included in the model, while the green line represents the results without genotype in the model. (PDF) [file pone.0051658.s003.pdf]

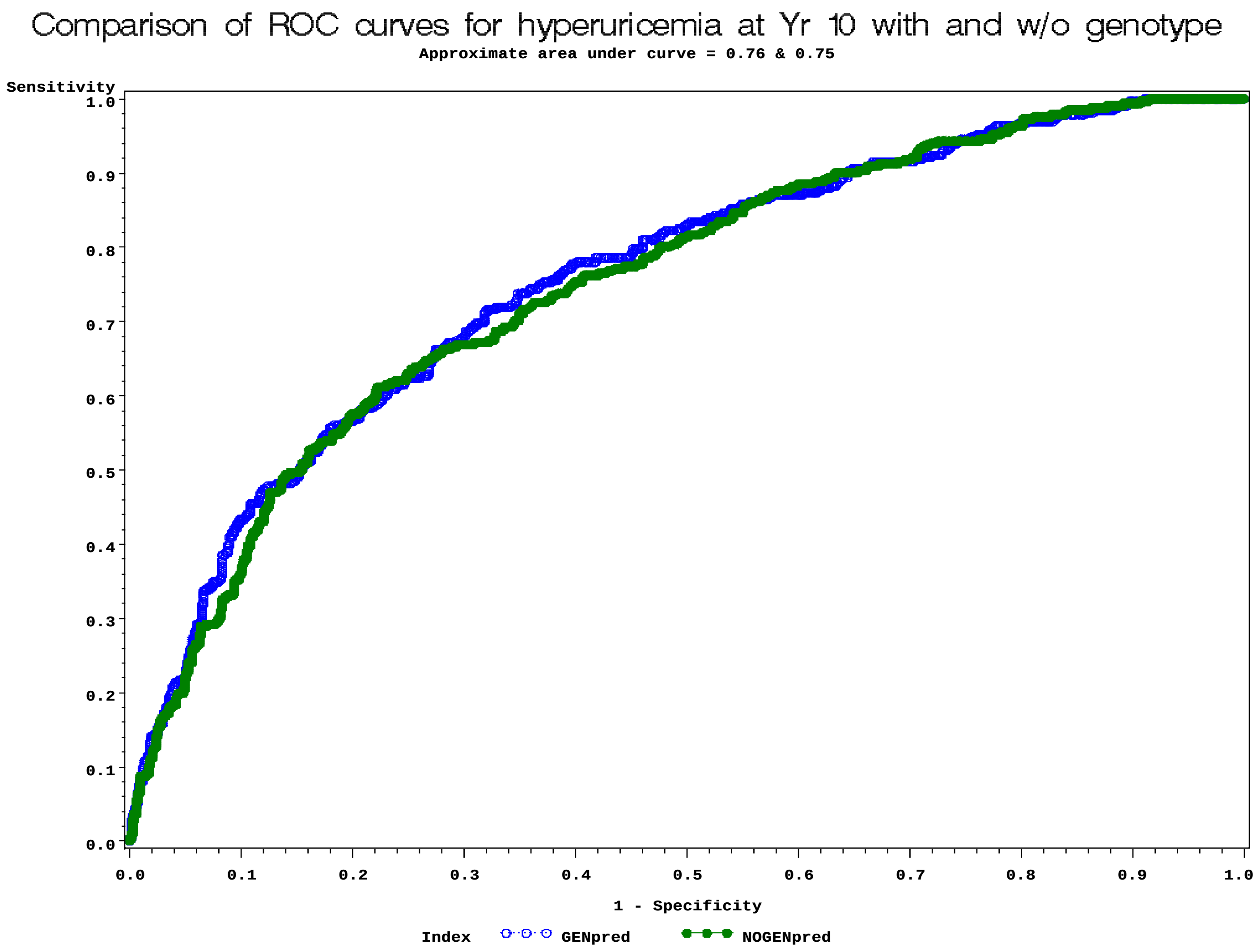


**Figure S3**. ROC curves for the prediction of prevalent hyperuricemia at Year 10 in SOS subjects with (blue line) and without (green line) the inclusion of SLC2A9 rs13113918 genotype in the model.
