# Supplementary material for: Changes in Uric Acid Levels following Bariatric Surgery Are Not Associated with SLC2A9 Variants in the Swedish Obese Subjects Study
Source: PLoS One. 2012 Dec 14;7(12):e51658. doi: 10.1371/journal.pone.0051658 (PMC3522707; doi:10.1371/journal.pone.0051658)
Supplement: Table S1 — Results of multivariate regression model with forward selection for predictors of change in uric acid from baseline to Year 2. (DOC) [file pone.0051658.s004.doc]

**Table S1**. Results of multivariate regression model with forward selection for predictors of change in uric acid from baseline to Year 2.

| **Variable** | **partial R^2^** | **Model R^2^** | **F value** | **P value** |
| --- | --- | --- | --- | --- |
| **Δweight (baseline-yr 2), kg** | 0.141 | 0.141 | 251.16 | <0.0001 |
| **Diabetes status** | 0.016 | 0.157 | 29.02 | <0.0001 |
| **Δtriglycerides (baseline-yr 2), mmol/L** | 0.013 | 0.170 | 22.90 | <0.0001 |
| **Sex, M vs F** | 0.001 | 0.171 | 1.40 | 0.2365 |
| **ΔHDL-C (baseline-yr 2), mmol/L** | 0.000 | 0.171 | 0.45 | 0.5004 |
| **Age, years** | 0.000 | 0.171 | 0.10 | 0.7468 |
| **Lipid medication status** | 0.000 | 0.171 | 0.03 | 0.8651 |
| **Smoking status** | 0.000 | 0.171 | 0.03 | 0.8716 |
| **Surgical procedure** | 0.000 | 0.171 | 0.01 | 0.9325 |

Diabetes status: 1=never, 2=corrected diabetes by yr 2, 3=became diabetic by yr 2, 4=diabetic at both baseline and yr 2.

Lipid medication status: 1= on lipid meds at either time point, 0=not on lipid meds at both time points

Smoking status: 1=never smoker, 2=quit smoking by yr 2, 3=began smoking by yr 2, 4=smoker at both baseline and yr 2.

Surgical procedure: 1=vertical banded gastroplasty, 2=banding procedures, 3=gastric bypass
