# Supplementary material for: Changes in Uric Acid Levels following Bariatric Surgery Are Not Associated with SLC2A9 Variants in the Swedish Obese Subjects Study
Source: PLoS One. 2012 Dec 14;7(12):e51658. doi: 10.1371/journal.pone.0051658 (PMC3522707; doi:10.1371/journal.pone.0051658)
Supplement: Table S2 — Results of multivariate regression model with forward selection for predictors of change in uric acid from Year 2 to Year 10. (DOC) [file pone.0051658.s005.doc]

**Table S2. Results of multivariate regression model with forward selection for predictors of change in uric acid from Year 2 to Year 10.**

| **Variable** | **partial R^2^** | **Model R^2^** | **F value** | **P value** |
| --- | --- | --- | --- | --- |
| **Δweight (yr 2-yr 10), kg** | 0.120 | 0.120 | 147.50 | <0.0001 |
| **Sex, M vs F** | 0.017 | 0.136 | 22.16 | <0.0001 |
| **Age, years** | 0.007 | 0.142 | 8.31 | 0.0040 |
| **Diabetes status** | 0.006 | 0.148 | 7.71 | 0.0056 |
| **Δtriglycerides (yr 2-yr 10), mmol/L** | 0.003 | 0.152 | 4.39 | 0.0364 |
| **Smoking status** | 0.001 | 0.153 | 0.99 | 0.3188 |
| **Lipid medication status** | 0.000 | 0.153 | 0.10 | 0.7525 |
| **ΔHDL-C (yr 2-yr 10), mmol/L** | 0.000 | 0.153 | 0.06 | 0.8106 |
| **Surgical procedure** | 0.000 | 0.153 | 0.01 | 0.9316 |

Diabetes status: 1=never, 2=corrected diabetes by yr 10, 3=became diabetic by yr 10, 4=diabetic at both yrs 2 and 10.

Lipid medication status: 1= on lipid meds at either time point, 0=not on lipid meds at both time points

Smoking status: 1=never smoker, 2=quit smoking by yr 10, 3=began smoking by yr 10, 4=smoker at both yrs 2 and 10.

Surgical procedure: 1=vertical banded gastroplasty, 2=banding procedures, 3=gastric bypass
