# Supplementary material for: Changes in Uric Acid Levels following Bariatric Surgery Are Not Associated with SLC2A9 Variants in the Swedish Obese Subjects Study
Source: PLoS One. 2012 Dec 14;7(12):e51658. doi: 10.1371/journal.pone.0051658 (PMC3522707; doi:10.1371/journal.pone.0051658)
Supplement: Table S3 — SNP physical map locations, minor allele frequencies (MAF), Hardy-Weinberg equilibrium (HWE) test statistics, and pairwise linkage disequilibrium estimates (r2 below median, D’ above median) for SLC2A9 SNPs in SOS bariatric surgery patients. (DOC) [file pone.0051658.s006.doc]

**Table S3**. SNP physical map locations, minor allele frequencies (MAF), Hardy-Weinberg equilibrium (HWE) test statistics, and pairwise linkage disequilibrium estimates (r2 below median, D’ above median) for SLC2A9 SNPs in SOS bariatric surgery patients.

| **Solute carrier family 2, member 9 (*SLC2A9*):** | | | | | |  |  |  |  |  |  |  |  |  |  |  |  |  |  |
| --- | --- | --- | --- | --- | --- | --- | --- | --- | --- | --- | --- | --- | --- | --- | --- | --- | --- | --- | --- |
| **SNP** | **Position (Chr 4)** | **Minor allele** | **MAF** | **HWE** | **Rank** | **1** | **2** | **3** | **4** | **5** | **6** | **7** | **8** | **9** | **10** | **11** | **12** | **13** | **14** |
| rs2280205 | 9,519,021 | G | 0.47 | 0.1544 | **1** |  | 0.68 | 0.49 | 0.40 | 0.46 | 0.62 | 0.56 | 0.54 | 0.54 | 0.56 | 0.52 | 0.44 | 0.67 | 0.63 |
| rs3733591 | 9,531,228 | A | 0.2 | 0.6473 | **2** | 0.11 |  | 1.00 | 0.33 | 0.86 | 0.63 | 0.98 | 0.96 | 0.96 | 0.95 | 0.96 | 0.27 | 0.80 | 0.95 |
| rs734553 | 9,532,102 | C | 0.23 | 0.23 | **3** | 0.08 | 0.08 |  | 0.98 | 0.98 | 1.00 | 0.96 | 0.95 | 0.95 | 0.94 | 0.94 | 0.79 | 0.95 | 0.10 |
| rs13129697 | 9,536,065 | C | 0.26 | 0.3578 | **4** | 0.06 | 0.01 | **0.82** |  | 0.91 | 0.98 | 0.95 | 0.95 | 0.95 | 0.94 | 0.94 | 0.94 | 0.96 | 0.20 |
| rs737267 | 9,543,842 | A | 0.24 | 0.9694 | **5** | 0.08 | 0.06 | **0.92** | 0.76 |  | 1.00 | 1.00 | 0.99 | 0.99 | 0.99 | 0.98 | 0.81 | 0.97 | 0.07 |
| rs4447863 | 9,548,067 | G | 0.49 | 0.6389 | **6** | 0.35 | 0.10 | 0.31 | 0.35 | 0.33 |  | 1.00 | 0.99 | 0.99 | 0.98 | 0.97 | 0.98 | 0.99 | 0.96 |
| rs7442295 | 9,575,478 | G | 0.21 | 0.857 | **7** | 0.09 | 0.06 | 0.78 | 0.66 | 0.80 | 0.26 |  | 1.00 | 1.00 | 0.99 | 0.85 | 0.85 | 0.98 | 0.12 |
| rs13131257 | 9,590,987 | A | 0.2 | 0.8795 | **8** | 0.08 | 0.06 | 0.73 | 0.62 | 0.75 | 0.25 | **0.93** |  | 1.00 | 0.93 | 0.84 | 0.84 | 0.88 | 0.08 |
| rs13125646 | 9,591,428 | A | 0.2 | 0.8613 | **9** | 0.08 | 0.06 | 0.73 | 0.62 | 0.75 | 0.25 | **0.93** | **1.00** |  | 0.93 | 0.84 | 0.84 | 0.88 | 0.08 |
| rs6449213 | 9,603,313 | G | 0.18 | 0.2487 | **10** | 0.08 | 0.05 | 0.66 | 0.56 | 0.68 | 0.22 | **0.85** | 0.79 | 0.79 |  | 0.99 | 0.99 | 0.98 | 0.05 |
| rs13113918 | 9,607,591 | A | 0.21 | 0.1517 | **11** | 0.08 | 0.06 | 0.75 | 0.64 | 0.77 | 0.25 | 0.73 | 0.67 | 0.67 | **0.86** |  | 1.00 | 0.98 | 0.03 |
| rs1014290 | 9,610,959 | G | 0.24 | 0.1856 | **12** | 0.07 | 0.01 | 0.62 | 0.78 | 0.63 | 0.30 | 0.61 | 0.56 | 0.56 | 0.72 | **0.84** |  | 1.00 | 0.07 |
| rs9291642 | 9,616,373 | G | 0.29 | 0.7073 | **13** | 0.07 | 0.02 | 0.43 | 0.38 | 0.43 | 0.15 | 0.54 | 0.46 | 0.46 | 0.62 | 0.54 | 0.47 |  | 1.00 |
| rs6820230 | 9,636,640 | A | 0.3 | 0.9386 | **14** | 0.18 | 0.10 | 0.00 | 0.01 | 0.00 | 0.39 | 0.00 | 0.00 | 0.00 | 0.00 | 0.00 | 0.00 | 0.06 |  |
