# Supplementary material for: Changes in Uric Acid Levels following Bariatric Surgery Are Not Associated with SLC2A9 Variants in the Swedish Obese Subjects Study
Source: PLoS One. 2012 Dec 14;7(12):e51658. doi: 10.1371/journal.pone.0051658 (PMC3522707; doi:10.1371/journal.pone.0051658)
Supplement: Table S4 — Associations between changes in serum uric acid levels and SLC2A9 SNPs in SOS patients by surgical procedure. (DOC) [file pone.0051658.s007.doc]

**Table S4**. Associations between changes in serum uric acid levels and *SLC2A9* SNPs in SOS patients by surgical procedure.

All models are adjusted for age, sex, and percent change in body weight. β values represent change in changes in uric acid level (µmol/L) per copy of minor allele carried. To convert µmol/L to mg/dL divide values by 59.48**.**
