# Supplementary material for: Changes in Uric Acid Levels following Bariatric Surgery Are Not Associated with SLC2A9 Variants in the Swedish Obese Subjects Study
Source: PLoS One. 2012 Dec 14;7(12):e51658. doi: 10.1371/journal.pone.0051658 (PMC3522707; doi:10.1371/journal.pone.0051658)
Supplement: Table S5 — Cross-sectional associations between serum uric acid levels and SLC2A9 SNPs in SOS vertical banded gastroplasty patients when number of subjects has been maximized locally. (DOC) [file pone.0051658.s008.doc]

**Table S5**. Cross-sectional associations between serum uric acid levels and SLC2A9 SNPs in SOS vertical banded gastroplasty patients when number of subjects has been maximized locally.

|  | **Baseline** | | | **Year 2** | | | **Year 10** | | |
| --- | --- | --- | --- | --- | --- | --- | --- | --- | --- |
| ***SLC2A9*** | **N=1213** | | | **N=1105** | | | **N=856** | | |
| **SNP** | **β** | **R^2^** | **p-value** | **β** | **R^2^** | **p-value** | **β** | **R^2^** | **p-value** |
| rs2280205 | -7.45 | 0.57% | 0.01 | -6.97 | 0.62% | 0.01 | -3.34 | 0.10% | 0.38 |
| rs3733591 | 1.60 | 0.02% | 0.65 | 1.67 | 0.02% | 0.62 | -4.98 | 0.14% | 0.29 |
| rs734553 | -25.15 | 4.73% | 5.3x10^-14^ | -20.03 | 3.75% | 1.7x10^-10^ | -23.93 | 3.63% | 4.40x10^-8^ |
| rs13129697 | -22.45 | 4.07% | 3.1x10^-12^ | -18.87 | 3.59% | 4.5x10^-10^ | -24.52 | 4.11% | 9.70x10^-9^ |
| rs737267 | -23.28 | 4.17% | 6.3x10^-13^ | -18.54 | 3.31% | 1.2x10^-9^ | -22.67 | 3.35% | 7.80x10^-8^ |
| rs4447863 | 13.87 | 1.97% | 1.1x10^-6^ | 11.65 | 1.74% | 1.2x10^-5^ | 12.93 | 1.45% | 0.00041 |
| rs7442295 | -26.07 | 4.66% | 4.6x10^-14^ | -21.58 | 3.99% | 3.2x10^-11^ | -20.44 | 2.43% | 6.90x10^-6^ |
| rs13131257 | -25.05 | 4.15% | 1.1x10^-12^ | -21.09 | 3.67% | 2.1x10^-10^ | -21.36 | 2.55% | 3.70x10^-6^ |
| rs13125646 | -24.90 | 4.10% | 1.5x10^-12^ | -21.04 | 3.66% | 2.2x10^-10^ | -21.38 | 2.56% | 3.60x10^-6^ |
| rs6449213 | -26.16 | 4.28% | 1.0x10^-12^ | -23.06 | 4.16% | 2.4x10^-11^ | -23.15 | 2.84% | 1.50x10^-6^ |
| rs13113918 | -26.61 | 4.85% | 2.6x10^-14^ | -21.95 | 4.13% | 2.5x10^-11^ | -28.09 | 4.58% | 8.00x10^-10^ |
| rs1014290 | -24.39 | 4.50% | 2.9x10^-13^ | -20.58 | 4.00% | 6.1x10^-11^ | -27.17 | 4.73% | 9.40x10^-10^ |
| rs9291642 | -19.96 | 1.90% | 1.9x10^-6^ | -18.91 | 2.13% | 1.6 x10^-6^ | -21.72 | 1.90% | 7.20x10^-5^ |
| rs6820230 | -4.05 | 0.14% | 0.19 | -2.83 | 0.09% | 0.33 | -1.76 | 0.02% | 0.65 |

All models are adjusted for age, sex, and body weight. β values represent change in cross-sectional uric acid level (µmol/L) per copy of minor allele carried. To convert µmol/L to mg/dL divide values by 59.48.
