# Supplementary material for: Changes in Uric Acid Levels following Bariatric Surgery Are Not Associated with SLC2A9 Variants in the Swedish Obese Subjects Study
Source: PLoS One. 2012 Dec 14;7(12):e51658. doi: 10.1371/journal.pone.0051658 (PMC3522707; doi:10.1371/journal.pone.0051658)
Supplement: Table S6 — Cross-sectional associations between serum uric acid levels and SLC2A9 SNPs in SOS banding patients when number of subjects has been maximized locally. (DOC) [file pone.0051658.s010.doc]

**Table S6**. Cross-sectional associations between serum uric acid levels and SLC2A9 SNPs in SOS banding patients when number of subjects has been maximized locally.

|  | **Baseline** | | | **Year 2** | | | **Year 10** | | |
| --- | --- | --- | --- | --- | --- | --- | --- | --- | --- |
| ***SLC2A9*** | **N=345** | | | **N=328** | | | **N=248** | | |
| **SNP** | **β** | **R^2^** | **p-value** | **β** | **R^2^** | **p-value** | **β** | **R^2^** | **p-value** |
| rs2280205 | 0.83 | 0.01% | 0.88 | 2.81 | 0.10% | 0.55 | -2.47 | 0.06% | 0.68 |
| rs3733591 | -5.72 | 0.21% | 0.41 | -1.49 | 0.02% | 0.81 | 3.47 | 0.09% | 0.65 |
| rs734553 | -22.03 | 3.08% | 0.001 | -21.41 | 3.93% | 4.6x10^-4^ | -12.98 | 1.19% | 0.10 |
| rs13129697 | -21.33 | 3.21% | 0.001 | -21.72 | 4.50% | 1.0x10^-4^ | -10.32 | 0.83% | 0.16 |
| rs737267 | -21.31 | 3.02% | 0.002 | -16.22 | 2.36% | 0.007 | -11.84 | 1.03% | 0.12 |
| rs4447863 | -5.98 | 0.34% | 0.03 | -3.17 | 0.13% | 0.052 | -11.33 | 1.36% | 0.07 |
| rs7442295 | -27.24 | 4.39% | 8.4x10^-5^ | -21.78 | 3.78% | 3.9x10^-4^ | -11.32 | 0.84% | 0.15 |
| rs13131257 | -26.24 | 3.90% | 2.0x10^-4^ | -20.47 | 3.20% | 0.001 | -12.64 | 1.00% | 0.12 |
| rs13125646 | -26.24 | 3.90% | 2.0x10^-4^ | -20.47 | 3.20% | 0.001 | -12.64 | 1.00% | 0.12 |
| rs6449213 | -28.03 | 4.39% | 5.1x10^-5^ | -20.77 | 3.26% | 7.3x10^-4^ | -10.96 | 0.74% | 0.17 |
| rs13113918 | -25.41 | 3.88% | 2.3x10^-4^ | -21.10 | 3.61% | 5.5x10^-4^ | -11.45 | 0.87% | 0.15 |
| rs1014290 | -24.96 | 4.20% | 1.6x10^-4^ | -23.42 | 4.99% | 5.9x10^-5^ | -10.18 | 0.78% | 0.18 |
| rs9291642 | -26.35 | 2.54% | 0.002 | -19.89 | 1.95% | 0.01 | -9.85 | 0.39% | 0.31 |
| rs6820230 | 1.93 | 0.03% | 0.75 | 5.41 | 0.32% | 0.30 | -9.69 | 0.83% | 0.14 |

All models are adjusted for age, sex, and body weight. β values represent change in cross-sectional uric acid level (µmol/L) per copy of minor allele carried. To convert µmol/L to mg/dL divide values by 59.48.
