# Supplementary material for: Changes in Uric Acid Levels following Bariatric Surgery Are Not Associated with SLC2A9 Variants in the Swedish Obese Subjects Study
Source: PLoS One. 2012 Dec 14;7(12):e51658. doi: 10.1371/journal.pone.0051658 (PMC3522707; doi:10.1371/journal.pone.0051658)
Supplement: Table S7 — Cross-sectional associations between serum uric acid levels and SLC2A9 SNPs in SOS gastric bypass patients when number of subjects has been maximized locally. (DOC) [file pone.0051658.s009.doc]

**Table S7**. Cross-sectional associations between serum uric acid levels and SLC2A9 SNPs in SOS gastric bypass patients when number of subjects has been maximized locally.

|  | **Baseline** | | | **Year 2** | | | **Year 10** | | |
| --- | --- | --- | --- | --- | --- | --- | --- | --- | --- |
| ***SLC2A9*** | **N=248** | | | **N=231** | | | **N=97** | | |
| **SNP** | **β** | **R^2^** | **p-value** | **β** | **R^2^** | **p-value** | **β** | **R^2^** | **p-value** |
| rs2280205 | -6.61 | 0.37% | 0.36 | -15.80 | 3.21% | 0.01 | 0.85 | 0.01% | 0.95 |
| rs3733591 | -2.98 | 0.05% | 0.72 | 10.54 | 0.96% | 0.13 | -9.83 | 0.48% | 0.49 |
| rs734553 | -37.40 | 8.51% | 1.8x10^-6^ | -27.69 | 7.17% | 1.9x10^-5^ | -17.50 | 1.67% | 0.23 |
| rs13129697 | -33.01 | 7.01% | 1.3x10^-5^ | -25.43 | 6.40% | 4.7x10^-5^ | -25.31 | 3.68% | 0.07 |
| rs737267 | -43.09 | 11.43% | 2.2x10^-8^ | -29.17 | 8.06% | 5.4x10^-6^ | -36.38 | 7.29% | 0.01 |
| rs4447863 | -20.68 | 3.59% | 0.003 | -21.70 | 6.08% | 2.3x10^-4^ | -24.65 | 4.56% | 0.05 |
| rs7442295 | -38.10 | 8.01% | 5.3x10^-6^ | -27.51 | 6.42% | 7.6x10^-5^ | -30.88 | 4.70% | 0.04 |
| rs13131257 | -38.42 | 7.90% | 6.2x10^-6^ | -28.30 | 6.59% | 6.0x10^-5^ | -38.07 | 6.94% | 0.01 |
| rs13125646 | -38.42 | 7.90% | 6.2x10^-6^ | -28.30 | 6.59% | 6.0x10^-5^ | -38.07 | 6.94% | 0.01 |
| rs6449213 | -38.20 | 7.38% | 3.7x10^-5^ | -31.18 | 7.56% | 5.1x10^-5^ | -42.22 | 8.06% | 0.01 |
| rs13113918 | -42.29 | 9.50% | 2.2x10^-6^ | -33.35 | 9.08% | 6.9x10^-6^ | -40.48 | 7.79% | 0.02 |
| rs1014290 | -33.63 | 6.59% | 4.4x10^-5^ | -26.38 | 6.23% | 1.1x10^-4^ | -35.27 | 6.48% | 0.02 |
| rs9291642 | -30.59 | 3.51% | 0.003 | -24.30 | 3.40% | 0.004 | -16.48 | 0.91% | 0.41 |
| rs6820230 | 1.76 | 0.02% | 0.83 | -8.08 | 0.68% | 0.24 | -11.72 | 0.83% | 0.39 |

All models are adjusted for age, sex, and body weight. β values represent change in cross-sectional uric acid level (µmol/L) per copy of minor allele carried. To convert µmol/L to mg/dL divide values by 59.48
